# Supplementary material for: Long-Term Indoor-Outdoor PM2.5 Measurements Using PurpleAir Sensors: An Improved Method of Calculating Indoor-Generated and Outdoor-Infiltrated Contributions to Potential Indoor Exposure
Source: Sensors (Basel). 2023 Jan 19;23(3):1160. doi: 10.3390/s23031160 (PMC9920798; doi:10.3390/s23031160)
Supplement: Supplementary file 1 [file sensors-23-01160-s001.zip › sensors-2132793-supplementary.pdf]

Supplement to

Long-Term Indoor-Outdoor $PM_{2.5}$ Measurements Using PurpleAir Sensors: An Improved Method of Calculating Indoor-Generated and Outdoor-Infiltrated Contributions to Potential Indoor Exposure

Lance Wallace [1,*] and Wayne Ott [2]

[1] Independent Researcher, 428 Woodley Way, Santa Rosa, CA 95409, USA
[2] Department of Civil and Environmental Engineering, Stanford University, 1008 Cardiff Lane, Redwood City, CA 94061, USA; wott1@stanford.edu
* Correspondence: lwallace73@gmail.com

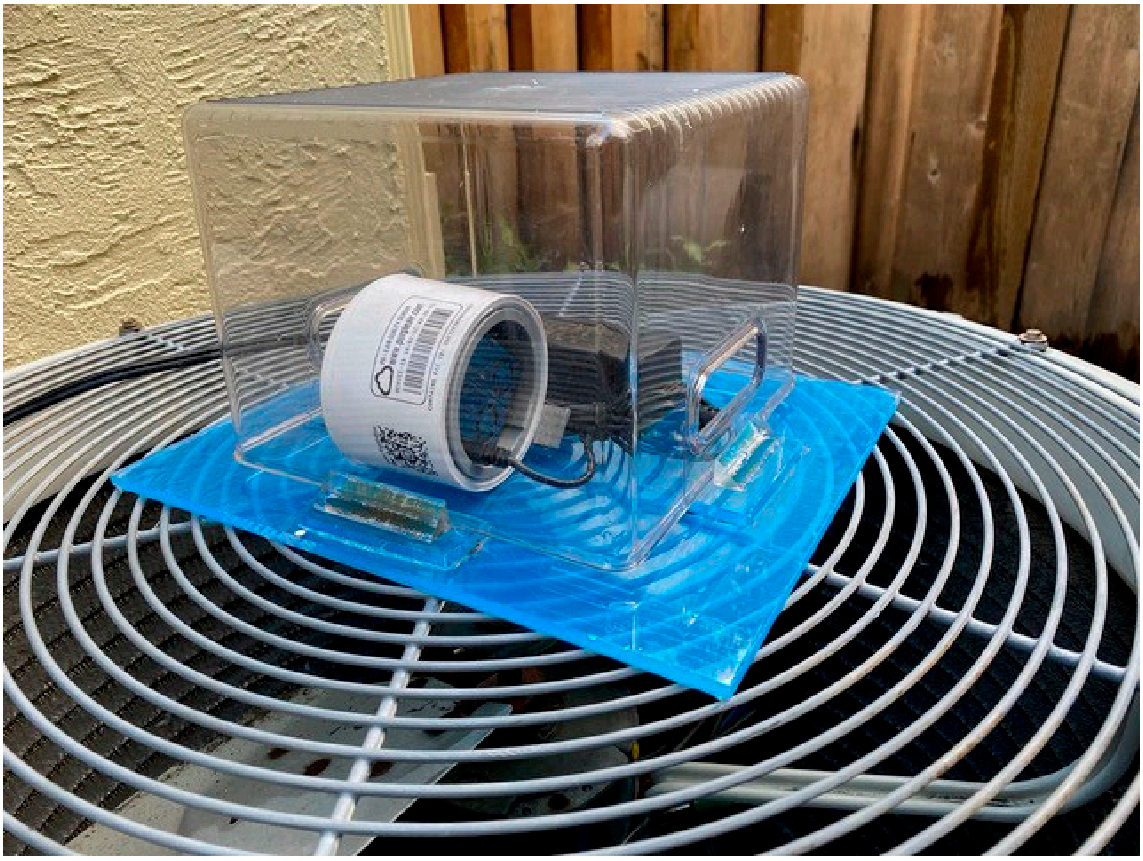

Figure S1. PurpleAir monitor setup for outdoor measurements.

Alexander Avenue

The I/O ratio for Alexander Avenue was plotted, but did not display the regular seasonal variations shown by the Oakmont and Redwood City I/O graphs in the main study (Figure S2).

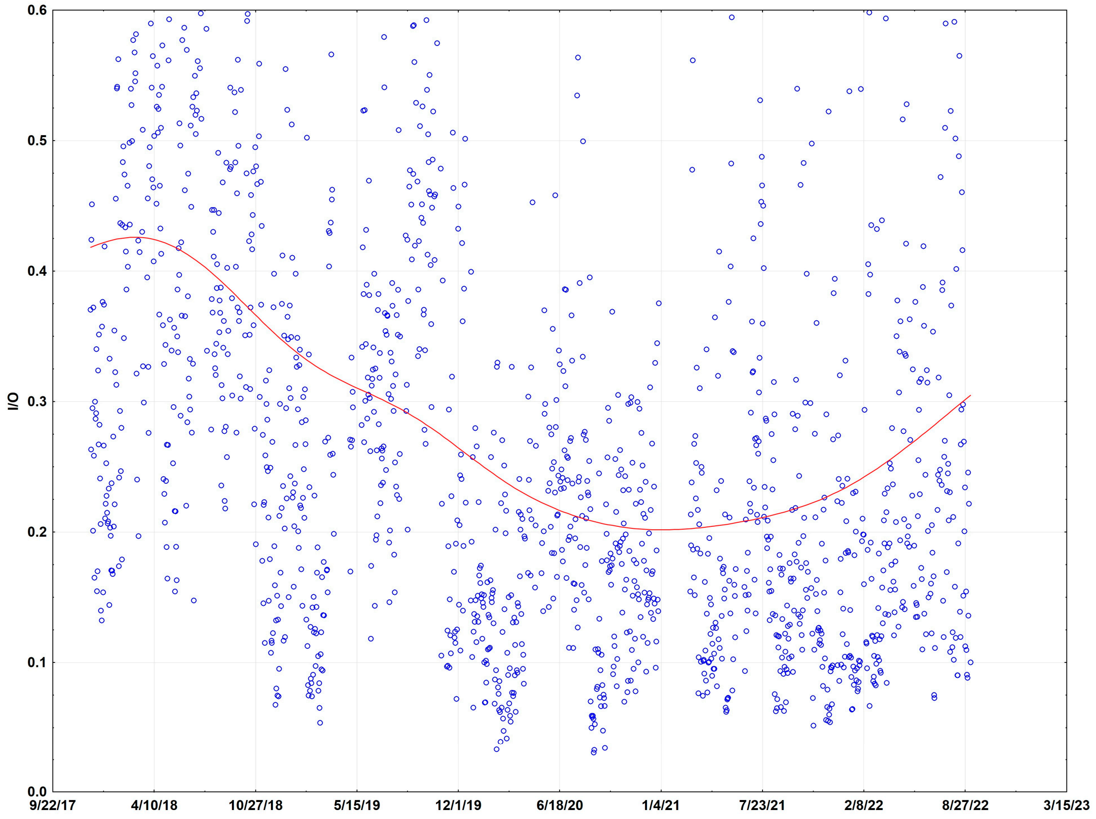

Figure S2. Indoor/Outdoor ratios for Alexander Avenue site, fitted by distance-weighted regression.

Bennett Valley

This outdoor site is about 4 km from the Oakmont site and was explored to see how the increased distance from the indoor monitor would affect the regression. Using the indoor/outdoor (I/O) ratio to have an initial idea of how to break up the full dataset, there were two troughs in winter, suggesting that that season would be good to investigate (Figure S3). Indeed ,in the associated full journal article, a good result was obtained using the December-June data (omitting March).

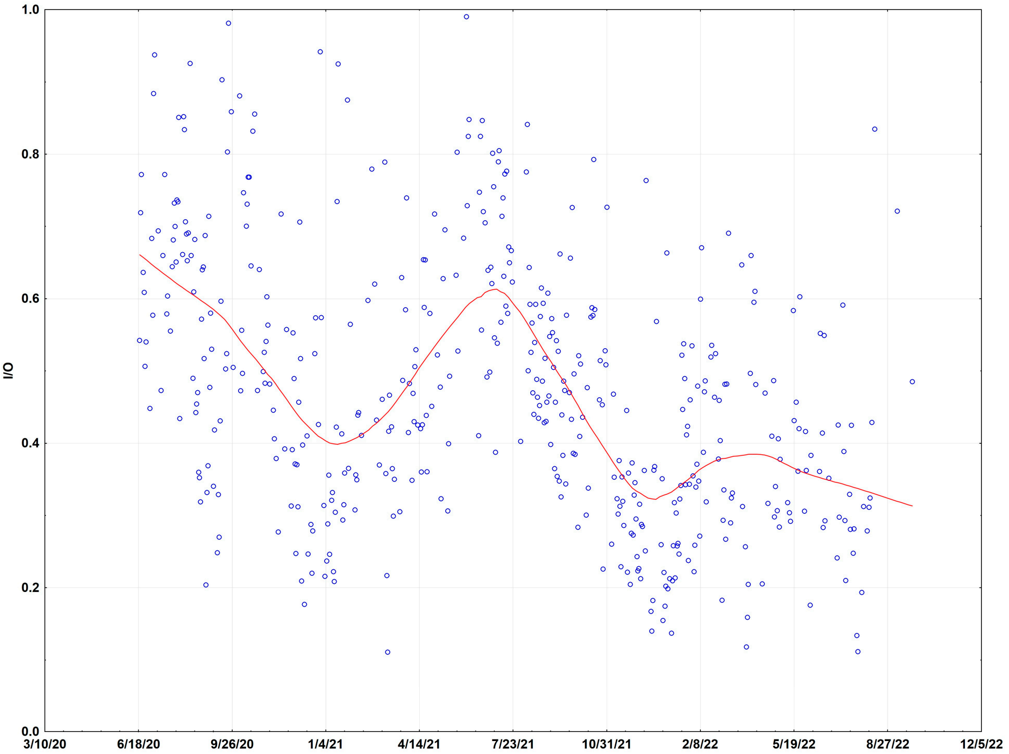

Figure S3. Indoor-outdoor (I/O) ratio of daily means at the Bennett Valley (outdoor) and Oakmont Santa Rosa (indoor) sites. Data are fit using distance-weighted regression.

The ALT-CF3 algorithm was developed to provide a transparent and reproducible alternative to the "black box" algorithms provided by the manufacturer (Plantower) of the sensors used in Purpleair monitors. It is described fully in Bi et al., (2021) and Wallace et al., (2021, 2022). The algorithm uses the particle numbers reported for the three size fractions 0.3-0.5 um, 0.5-1 um, and 1-2.5 um. By assigning an average diameter to each size fraction, the total particle volume is calculated. The diameter chosen could be, for example, the arithmetic or geometric mean of the upper and lower boundaries of the size category. In our case, we chose the geometric mean, also used by a major manufacturer of multichannel optical particle counters (TSI). The total PM2.5 mass is then determined in two steps: first a density is assigned (in this case the density of water) and then a calibration factor is determined by comparison to nearby regulatory monitors employing Federal Reference or Federal Equivalent Methods (FRM/FEM). The original calibration factor for 33 PurpleAir PA-II monitors within 0.5 km of 27 regulatory monitors was 3.0, leading to naming this alternative algorithm ALT-CF3 (Wallace et al, 2021). More recently, a much wider selection of monitors in three states were used to further develop calibration factors for both the PA-I (indoor) and PA-II (outdoor) monitors (Wallace et al., 2022). Both calibration factors were identical at 3.4 for the PMS 1003 and PMS 5003 sensors. Since the value of 3.0 was determined for California monitors, we continue to use the value of 3 for the following three California sites. In the following tables, we refer to the ALT-CF3 algorithm as "CF3" and the Plantower CF_1 algorithm as "CF1".

Menlo Park

This site has one outdoor PA-II monitor (with two Plantower PMS 5003 sensors) and one indoor PA-I monitor (with one Plantower PMS 1003 sensor). The monitors operated from 11/13/2019 to 12/4/2021. The site is a private residence occupied by a renter. Between about July 2, 2020 and Feb 14, 2021, the renter was out of the country and the residence was unoccupied. This offered an interesting opportunity to compare indoor concentrations and indoor-outdoor relations in an occupied and unoccupied residence.

The period when the residence was unoccupied between 7/2/20 and 2/14/21 was marked by a total absence of indoor concentrations above about 20 µg/m^3, except for three major nearby wildfires, easily visible in the indoor record (Figure S4).

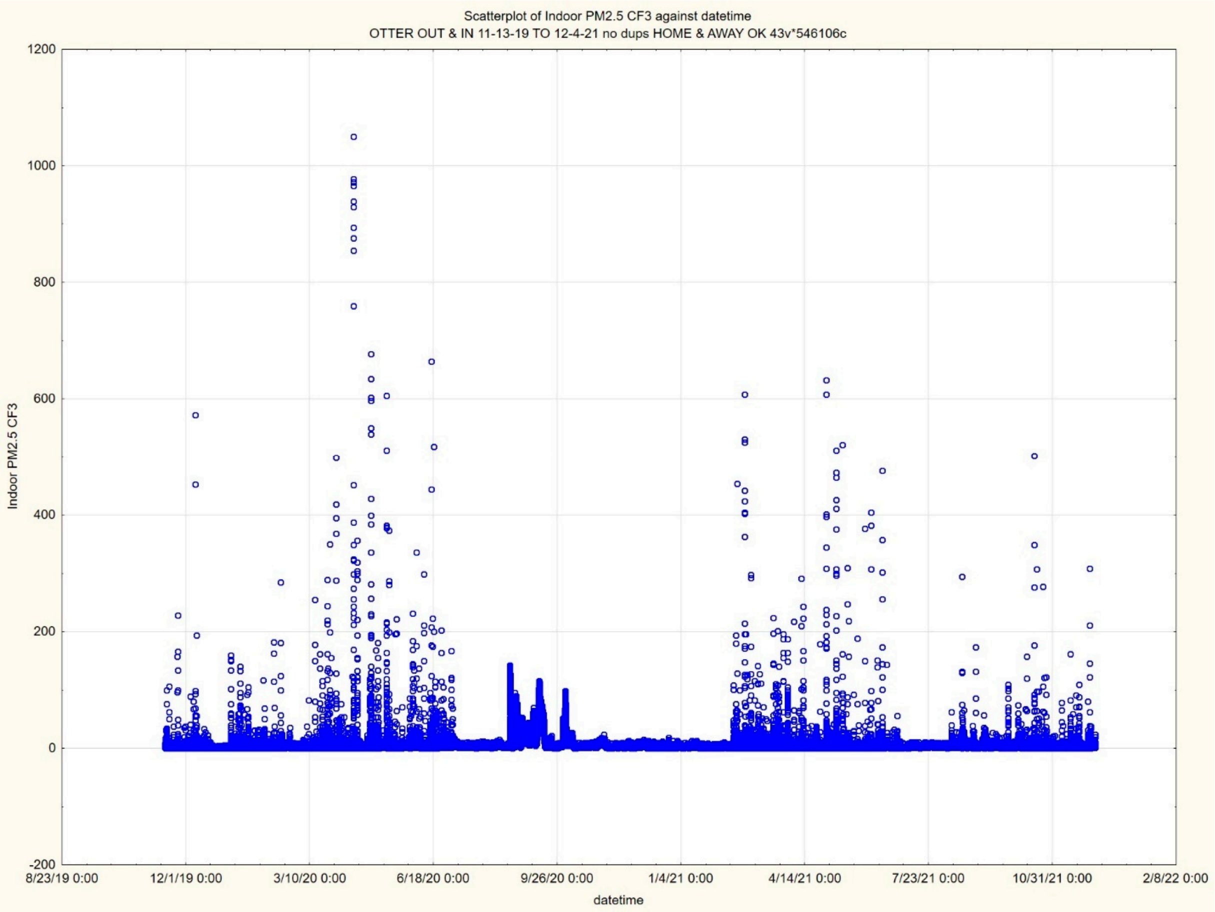

Figure S4. Indoor concentrations at Menlo Park site.

The indoor-generated concentrations during the time the house was occupied are shown in Figure S5. Many of these lasted for short times of 10-15 minutes.

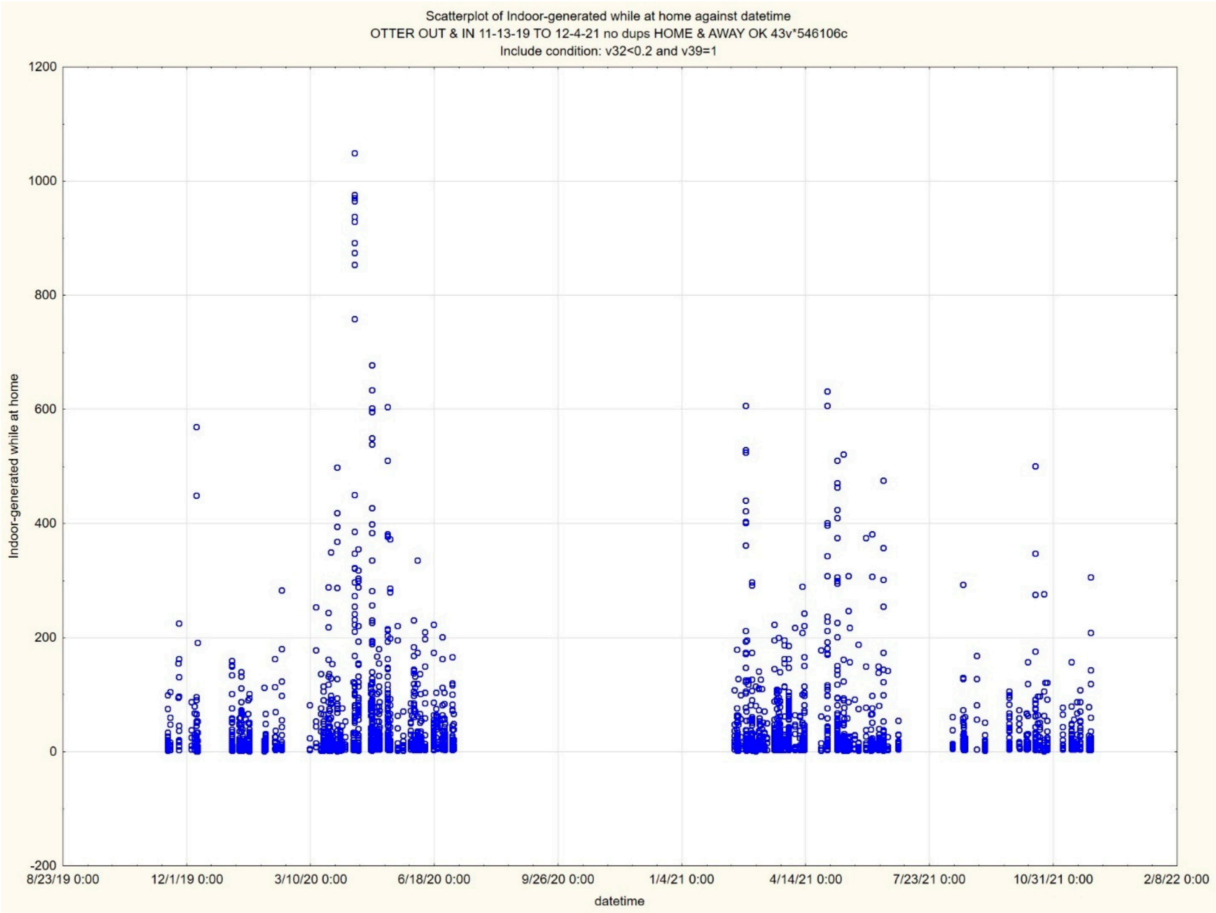

Figure S5. Indoor concentrations identified as due to indoor activities.

Outdoor concentrations are shown in Figure S6. The three peaks in August-September, 2020 are due to wildfires.

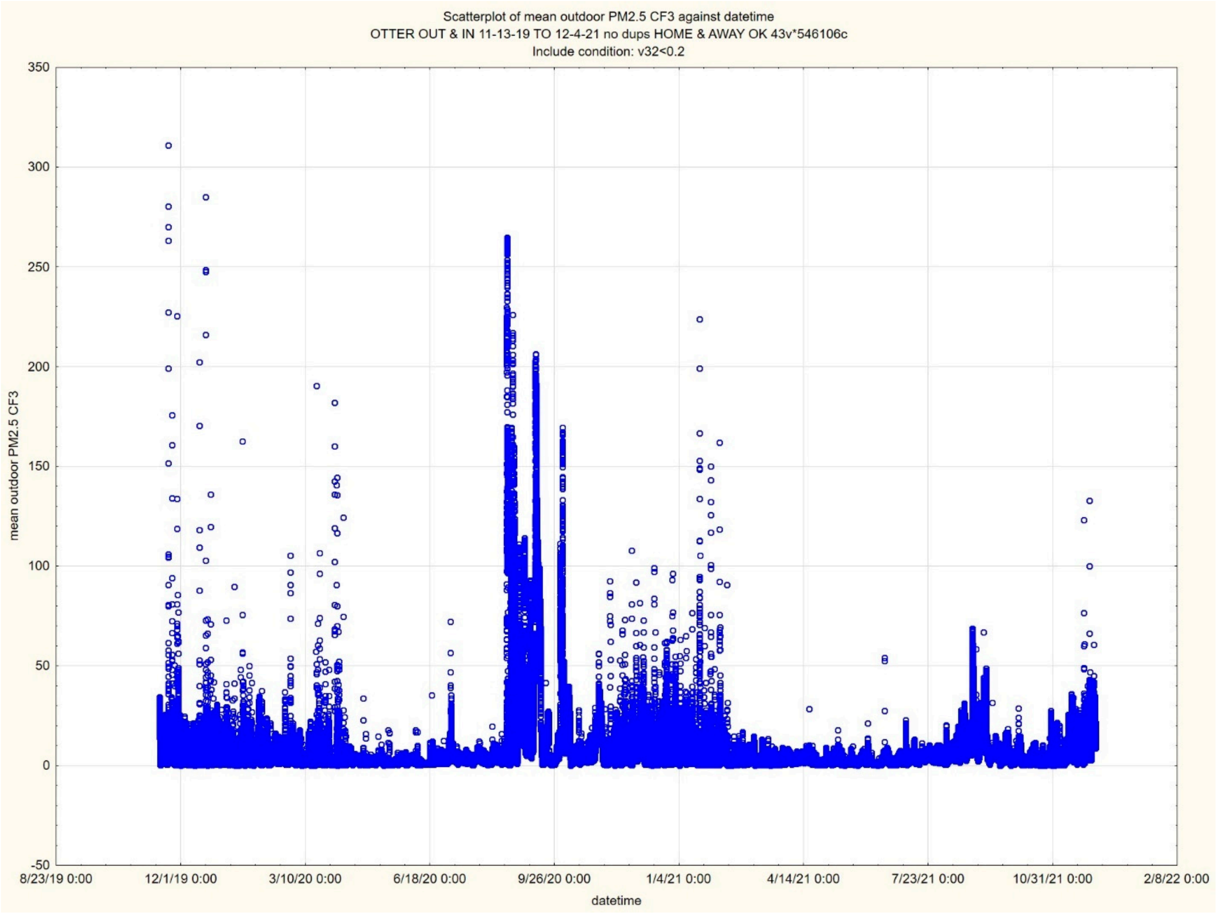

Figure S6. Outdoor $PM_{2.5}$ concentrations from 11/13/19 to 12/4/21.

When the house was unoccupied, the daily average values showed (as expected) no positive indoor-generated mean; in fact the results showed a (non-significant) negative mean value (intercept) of -0.92 µg/m^3 (Figure S7). In this case, there is no Forbidden Zone. However, the R^2 value of 0.96 suggests that the resulting infiltration factor of 0.68 is reasonably robust.

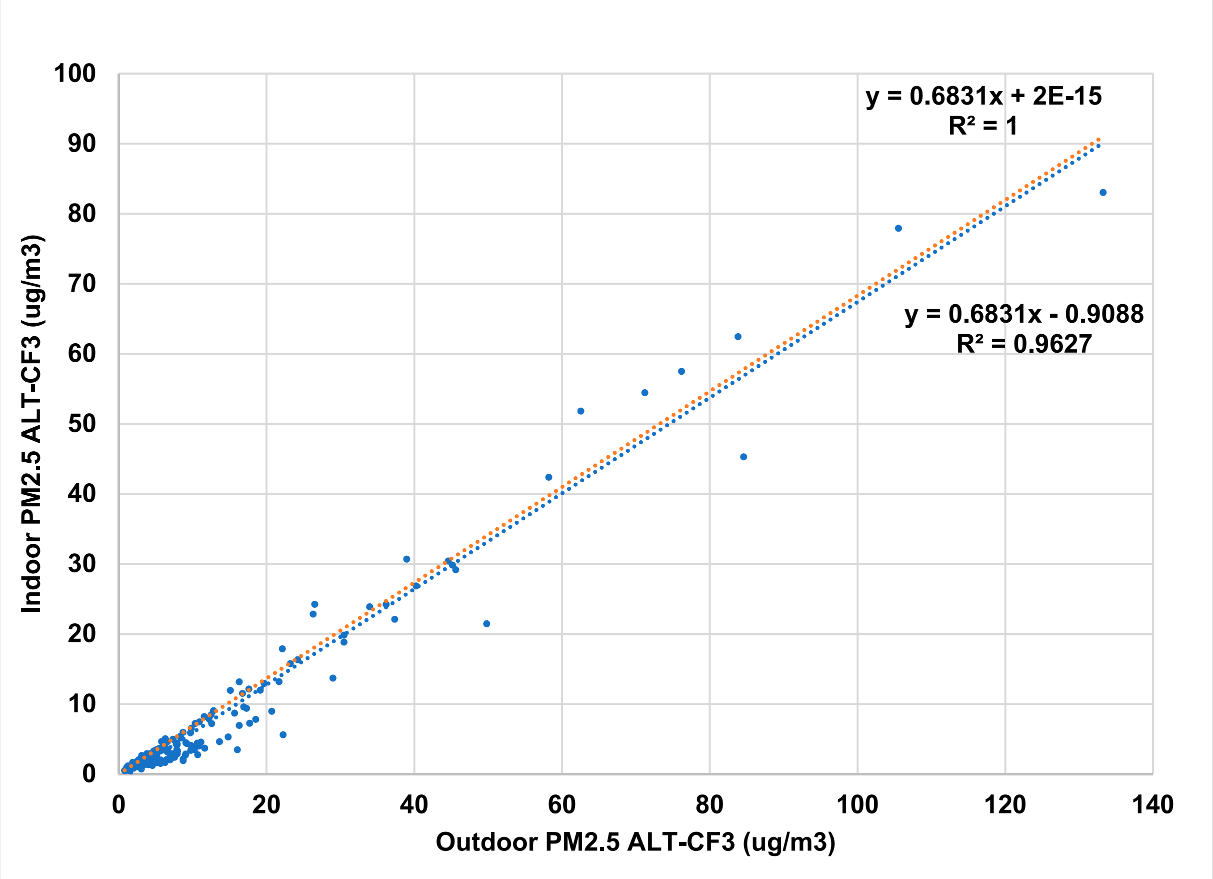

Figure S7. Regression of daily outdoor $PM_{2.5}$ on indoor $PM_{2.5}$ during the time the residence was unoccupied (7/9/2020 to 2/14/2021).

The data for the time of occupancy (270 days) consist of 2-minute average PM2.5 concentrations (Table S1). Note the loss of about 70,000 data points for the Plantower CF1 algorithm (bottom three rows) compared to the ALT-CF3 algorithm (top five rows). This is partly due to the Plantower decision to assign values of zero to concentrations below an arbitrary cutoff. No values of zero ever occur in the ALT-CF3 algorithm, since there are never occasions when the number of particles between 0.3 and 0.5 um are zero. The CF1 algorithm overestimates the outdoor PM2.5 concentration by about 85% (8.8 vs. 4.4 ug/m3) and the indoor concentration by 50% (3.3 vs. 2.2 ug/m3). The mean $PM_{2.5}$ concentration due to indoor activities (1.4 $\mu g/m^3$) is larger than the mean concentration (0.78 $\mu g/m^3$) attributable to penetration of ambient $PM_{2.5}$. The Spearman correlation coefficient was reasonably high at 0.67.

Table S1. $PM_{2.5}$ ($\mu g/m^3$) during the time of occupancy.

	N obs	Mean
Mean outdoor $PM_{2.5}$ CF3	372621	4.4
Indoor $PM_{2.5}$ CF3	369610	2.2
Outdoor-infiltrated CF3	372621	0.78
Indoor-generated CF3	369610	1.4

Precision outdoor CF3	372621	0.06
Mean outdoor CF1	304345	8.3
Indoor $PM_{2.5}$_CF1	301625	3.3
Precision outdoor CF1	304345	0.07

For the time the residence was unoccupied, virtually all of the indoor $PM_{2.5}$ was supplied by the outdoor air (mean of 7.0 µg/m^3 infiltrated out of a total mean of 7.2 µg/m^3) (Table S2). The mean indoor and outdoor PM2.5 concentrations were overestimated by 70% and 65% using the Plantower CF1 algorithm. Mean precision was also worse by about 75% using the CF1 algorithm.

Table S2. $PM_{2.5}$ (µg/m^3) during the time resident was out of the country.

	N obs	Mean	Std. Err.	Lower quartile	Geom. mean	Median	Upper quartile	Max
Mean outdoor CF3	145033	12	0.057	2.5	5.4	4.9	11	265
Indoor CF3	143876	7.2	0.037	1.5	3.1	2.8	5.6	142
Precision outdoor CF3	145033	0.051	0.00010	0.020	0.033	0.041	0.071	0.20
Outdoor-infiltrated CF3	145033	7.0	0.034	1.5	3.2	2.9	6.7	155
Indoor-generated CF3	143876	0.22	0.015	-0.82		0.085	0.83	64
Mean outdoor CF1	145028	20	0.10	3.5		7.8	20	448
Indoor CF1	143877	12	0.065	1.8		4.0	9.2	244
Precision outdoor CF1	143609	0.088	0.00034	0.025		0.056	0.10	1

The Spearman coefficient was quite high at 0.85 when the resident was out of the country.

Limit of detection (LOD)

Using the approach to calculate the LOD described in Wallace et al., 2010, the ALT-CF3 algorithm resulted in an estimated LOD of 1.24 µg/m^3 for the time the residence was unoccupied. There were 16001 (10.7%) $PM_{2.5}$ CF3 values below that concentration. For the CF1 algorithm, the LOD was 3.38 µg/m^3 and there were 38242 (25.7%) values below the LOD.

While the residence was occupied, the ALT-CF3 LOD was 1.10 µg/m^3, with 73458 (16.6%) $PM_{2.5}$ CF3 values below the LOD. For the CF1 algorithm, the LOD was 4.15 µg/m^3 and there were 215324 (48.6%) values below the LOD.

Lag times

An advantage of working with the raw 2-minute average data is the ability to estimate the lag times corresponding to the highest correlation between indoor and outdoor $PM_{2.5}$. Hourly average data used in many studies limits the resolution of the lag times to one hour (0, 1, 2 hours etc.). Here we choose a resolution of 10 minutes, although we could attempt an even finer resolution down to 2 minutes. The lag time is inversely related to the air exchange rate. We therefore expect a larger air exchange rate (smaller lag time) in an occupied residence with persons going in and out of doors than in the same residence closed up with no occupants. The two cases (occupied/not occupied) show a difference of almost a factor of 4 (30 minutes vs. 110 minutes) in the lag times with the highest Spearman correlations (Table S3).

Table S3. Spearman correlations (r_S) of indoor and outdoor $PM_{2.5}$ CF3 for lag times when residence was occupied or unoccupied

Residence Occupied	rs
Indoor $PM_{2.5}$ CF3 (no lag time)	0.65875
Indoor lag 20 min	0.66354
Indoor lag 30 min	0.66368
Indoor lag 40min	0.66263
Indoor lag 60 min	0.65535
Residence Unoccupied	
Indoor $PM_{2.5}$ CF3 (no lag time)	0.85159
lag indoor 60 min	0.89241
lag indoor 70 min	0.89556
lag indoor 80 min	0.89806
lag indoor 90 min	0.89985
lag indoor 100 min	0.90098
lag indoor 110 min	0.90153
lag indoor 120 min	0.90140
lag indoor 130 min	0.90034

Redwood City

A site in Redwood City, CA operated two PurpleAir PA-II monitors indoors and one PurpleAir PA-II monitor outdoors from July 3, 2020 until Feb 6, 2022 (~9 months). The 2-minute average indoor data for the full 19-month period are provided for both the Plantower CF1 and ALT-CF3 algorithms in Table S4. The PLantower CF1 algorithm again overestimated PM2.5 by about 50%.

Table S4. All indoor $PM_{2.5}$ data (µg/m^3) and associated precision compared for the ALT-CF3 and Plantower CF1 algorithms for the 19-month period 7/3/20 to 2/6/22

	N obs.	Mean	Std.Err.	Lower quartile	Median	Upper quartile	Max
ALT-CF3 algorithm							
1a $PM_{2.5}$ CF3	662753	4.3	0.020	0.99	1.9	3.5	735
1b$PM_{2.5}$ CF3	662739	4.1	0.021	0.87	1.7	3.2	736
2a $PM_{2.5}$ CF3	662589	3.9	0.017	0.85	1.7	3.2	520
2b $PM_{2.5}$ CF3	662607	4.2	0.018	0.96	1.9	3.6	558
Mean 1 CF3	662773	4.2	0.020	0.93	1.8	3.3	735
Mean 2 CF3	662635	4.0	0.017	0.92	1.8	3.4	539
Plantower CF1 algorithm							
1a $PM_{2.5}$_CF1	662767	6.4	0.032	0.79	2.4	5.3	1115
1b $PM_{2.5}$_CF1	662748	6.3	0.034	0.68	2.2	4.9	1184
2a $PM_{2.5}$_CF1	662566	5.8	0.028	0.60	2.0	4.7	839
2b $PM_{2.5}$_CF1	662610	6.4	0.030	0.76	2.5	5.5	928

Applying an upper bound of 20% to the precision resulted in about 8% of the $PM_{2.5}$ CF3 data being lost from each monitor, or 15% from the overall mean, compared to 27-33% of the Plantower CF1 data lost from the individual monitors and 40% from the overall mean (Table S5).

Table S5. All indoor $PM_{2.5}$ data (μg/m^3) and associated precision (upper limit 0.2) from 7/3/19 to 2/6/22

	N obs.	Mean	Std.Err.	Lower quartile	Median	Upper quartile	Max
ALT-CF3 algorithm							
1a $PM_{2.5}$ CF3	612076	4.6	0.022	1.1	2.0	3.7	735
1b$PM_{2.5}$ CF3	612076	4.3	0.023	1.0	1.8	3.4	736
precision 1	612076	0.072	6.4E-05	0.030	0.063	0.106	0.2
2a $PM_{2.5}$ CF3	608251	3.9	0.018	0.9	1.8	3.3	520
2b $PM_{2.5}$ CF3	608251	4.4	0.019	1.0	2.0	3.7	558
precision 2	608251	0.079	6.3E-05	0.040	0.074	0.113	0.2
Mean $PM_{2.5}$ CF3	566588	4.6	0.022	1.1	2.1	3.7	589
precision 1	566588	0.071	6.6E-05	0.029	0.062	0.105	0.2
precision 2	566588	0.079	6.4E-05	0.040	0.074	0.112	0.2
CF1 algorithm							
1a $PM_{2.5}$_CF1	484890	8.5	0.044	1.8	3.7	6.8	1115
1b $PM_{2.5}$_CF1	484890	8.4	0.047	1.7	3.4	6.3	1184
precision 1 CF1	484890	0.070	7.3E-05	0.027	0.059	0.105	0.2
2a $PM_{2.5}$_CF1	446292	7.9	0.040	1.7	3.3	6.2	839
2b $PM_{2.5}$_CF1	446292	9.0	0.044	1.9	3.9	7.4	928
precision 2 CF1	446292	0.091	7.6E-05	0.052	0.089	0.129	0.2
Mean $PM_{2.5}$ CF1	394818	9.6	0.051	2.3	4.1	7.4	946
precision 1 CF1	394818	0.066	7.9E-05	0.026	0.055	0.098	0.2
precision 2 CF1	394818	0.091	7.9E-05	0.052	0.088	0.128	0.2

Statistics for the outdoor data are compared for theAlt-CF3 and Plantower CF1 algorithms (Table S6). The first set of comparisons is for all data (>200,000 observations) and no limits on the precision. The second set of comparisons shows that applying a precision cutoff of 0.2 for the CF3 data results in a loss of about 14% of the data. The third set shows that applying the precision cutoff to the Plantower CF1 dataset results in a loss of 35% of the data.

Table S6. All outdoor $PM_{2.5}$ data (µg/m^3) and associated precision (upper limit 0.2) from 4/29/21 to 2/6/22

Sensor & algorithm ID	Valid N	Mean	Std. Err.	Lower quartile	Median	Upper quartile	Max
No precision cutoff							
3a $PM_{2.5}$ CF3	201291	7.0	0.026	1.8	4.1	8.6	840
3b $PM_{2.5}$ CF3	201294	7.1	0.025	2.3	4.5	8.6	822
precision CF3	201241	0.1	2.8E-04	0.026	0.058	0.13	1
3a $PM_{2.5}$_CF1	201276	9.7	0.040	1.2	5.0	12.2	1238
3b $PM_{2.5}$_CF1	201270	11.3	0.040	3.1	6.8	13.8	1199
precision CF1	201050	0.3	7.2E-04	0.056	0.16	0.44	1
CF3 precision <0.2							
3a $PM_{2.5}$ CF3	172543	8.0	0.028	2.6	5.0	9.8	840
3b $PM_{2.5}$ CF3	172543	8.0	0.028	2.9	5.3	9.6	822
precision CF3	172543	0.1	1.2E-04	0.022	0.048	0.087	0.2
3a $PM_{2.5}$_CF1	172500	11.2	0.044	2.4	6.4	14.2	1238
3b $PM_{2.5}$_CF1	172499	12.8	0.044	4.2	8.3	15.7	1199
precision CF1	172451	0.2	5.4E-04	0.046	0.12	0.28	1
CF1 precision<0.2							
3a $PM_{2.5}$ CF3	112885	11.1	0.042	5.1	7.8	13.7	840
3b $PM_{2.5}$ CF3	112894	10.9	0.041	5.2	7.8	13.2	822
precision CF3	112867	0.0	1.1E-04	0.016	0.034	0.057	1
3a $PM_{2.5}$_CF1	112915	16.2	0.064	6.5	10.9	20.5	1238
3b $PM_{2.5}$_CF1	112915	17.6	0.065	8.0	12.5	21.7	1199
precision CF1	112915	0.1	1.7E-04	0.028	0.065	0.118	0.2

Although the 3a and 3b sensors are in very good agreement for the CF3 algorithm, (mean values differing by only a few percent), for an unknown reason there is a consistent relative additive bias of about 1.5 µg/m^3 for the 3b sensor compared to the 3a sensor using the Plantower CF1 algorithm (Figure S8). The bias is almost completely additive, since the slope in an x-y plot of the two sensors is 1.0056, with an intercept of 1.5761 µg/m^3 (R^2 = 99%). This is a mystifying effect that we have not seen in many other datasets. This may constitute another reason for mistrust of the "black box" nature of the proprietary algorithms supplied by Plantower.

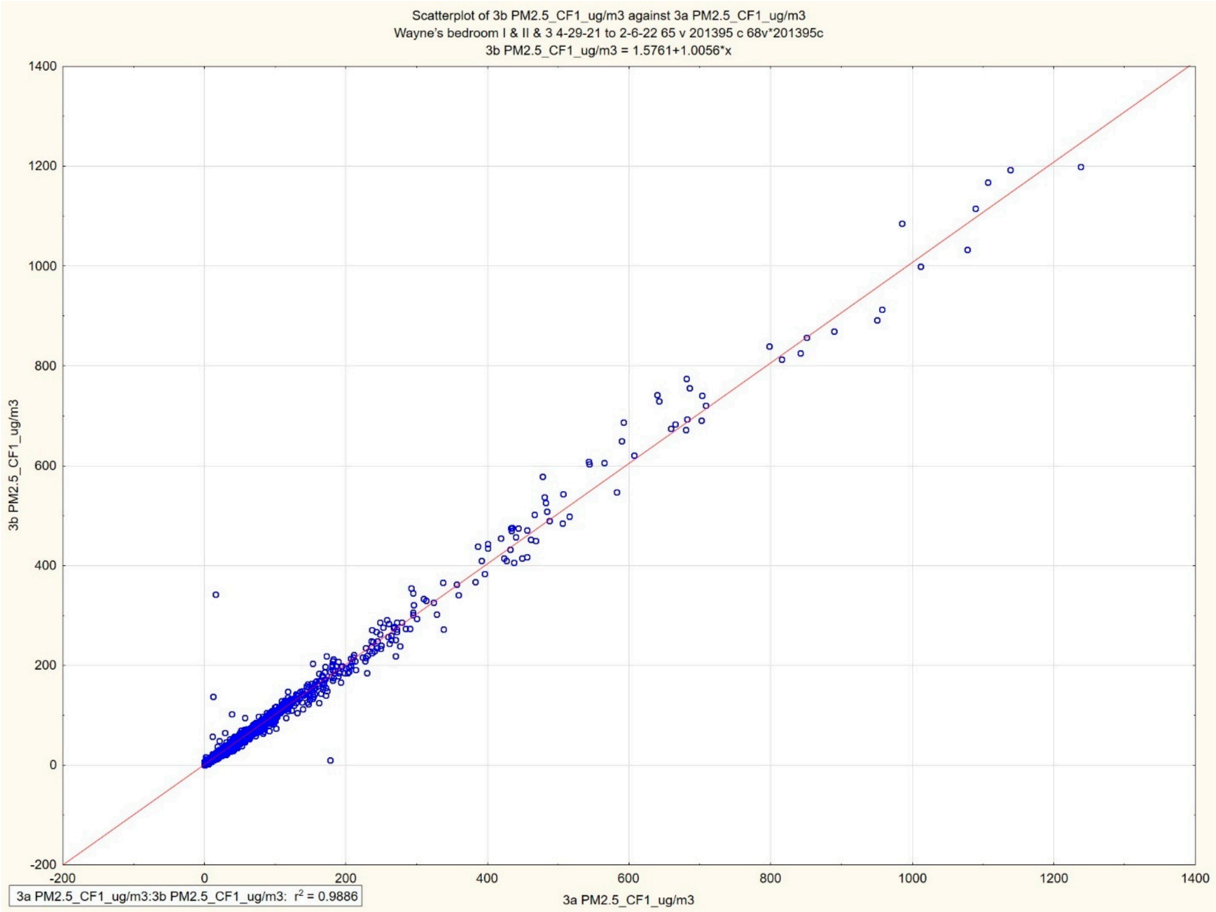

Figure S8. Outdoor Air $PM_{2.5}$ using the Plantower CF1 algorithm measured by sensor 3b is regressed against sensor 3a. The result shows a relative additive bias of 1.567 µg/m^3 coupled with an almost perfect absence of multiplicative bias (slope of 1.0056). This unusual feature is unexplained.

The extreme loss of data from use of the Plantower CF1 algorithm is largely due to the practice of setting all $PM_{2.5}$ values falling below a cutoff to zero. The number of zeros reported by the CF1 algorithm is shown for the 19-month dataset including all indoor data along with the 9-month dataset with outdoor data (Table S7). It is not possible to recover from this practice using any model or conversion factor based on the Plantower CF1 (or CF_ATM) algorithms. No zeros are ever reported by the CF3 algorithm, since particles are always present in the 0.3-0.5 um size category.

Table S7. Number of zeros reported for $PM_{2.5}$ values estimated using the Plantower CF1 algorithm.

	$PM_{2.5}$ values reported as zero using the CF1 algorithm in 9-month indoor-outdoor dataset	Fraction	$PM_{2.5}$ values reported as zero using the CF1 algorithm in 19-month dataset with only indoor air monitors	Fraction
1a CF1	15927	0.08	49013	0.07
1b CF1	18988	0.09	55532	0.08
2a CF1	22892	0.11	72442	0.11
2b CF1	17043	0.08	54864	0.08
3a CF1	20153	0.10		
3b CF1	185	0.00092		

The outlier value for the 3b (outdoor) sensor of only a tiny number of zeros has been checked from the original downloads and confirmed. It would be a natural result of adding 1.5 $\mu g/m^3$ to every datapoint. Because of the opaque nature of the proprietary Plantower algorithm, it is impossible to check on how this might have occurred. But it is another indication of how the lack of information about the Plantower CF1 algorithm can cause problems.

Indoor-outdoor relationship

A regression of the raw 2-minute average indoor $PM_{2.5}$ on outdoor $PM_{2.5}$ using the CF3 algorithm resulted in an estimated infiltration factor of 0.1384 and a mean value of 2.6815 µg/m^3 due to indoor-generated $PM_{2.5}$ (Figure S9).

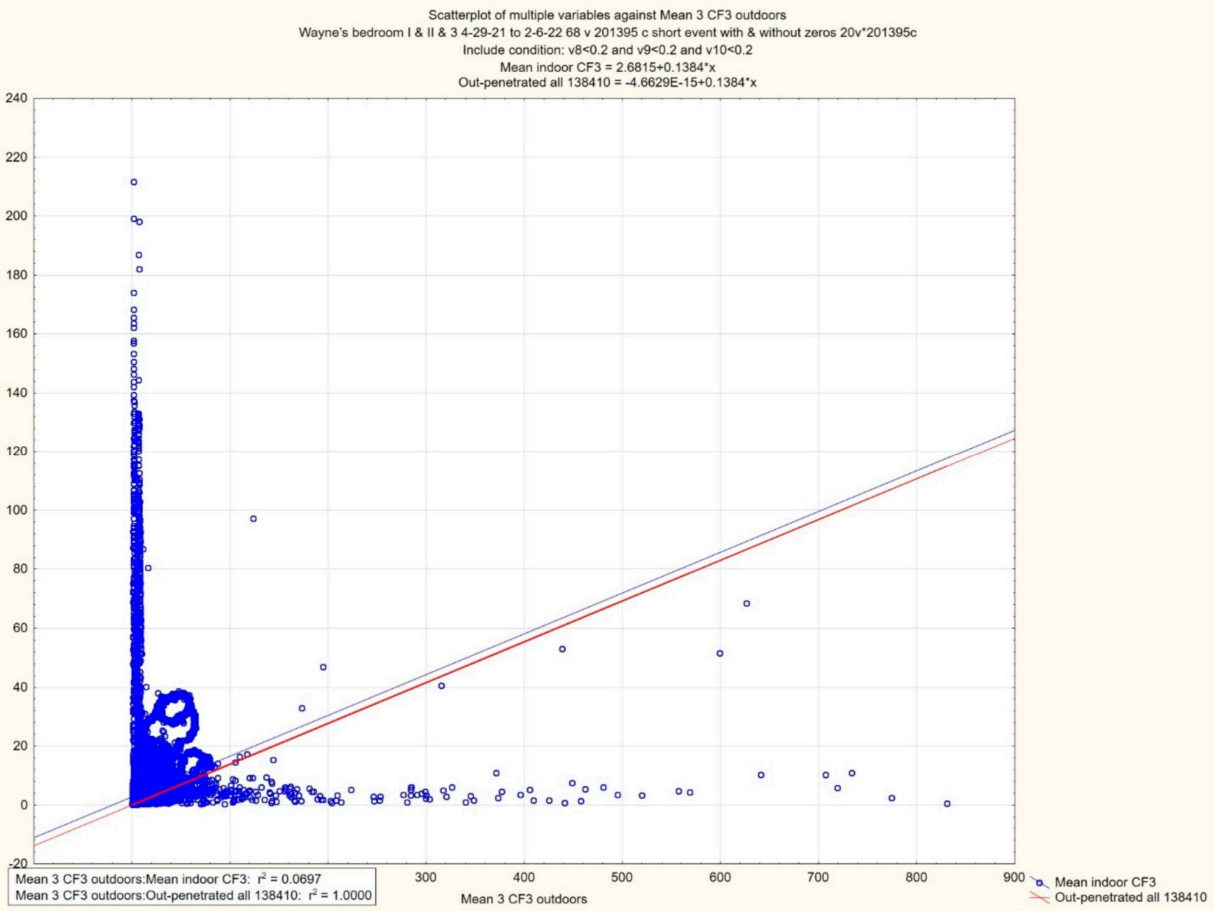

Figure S9. Regression of the raw 2-minute average indoor $PM_{2.5}$ data using the CF3 algorithm on outdoor $PM_{2.5}$ data. Precision of all data was required to be under 0.2.

There are many observations in the "forbidden" zone, suggesting that the assumption of a constant infiltration factor is false. The monthly regressions were found to support the idea of breaking the data into two datasets, since 7 months had low slopes whereas 2 months (August and September) had an average slope more than twice as high as the other 7 months (Table S8; Figure S10). Summer months may lead to higher air exchange rates due to opening windows.

Table S8. Regression statistics for the 7-month and 2-month periods.

	7 months		Aug-Sept	
	Intercept	slope	Intercept	slope
Mean	2.28	0.056	3.0	0.139
SD	0.75	0.031	1.0	0.048
SE	0.25	0.010	0.7	0.034

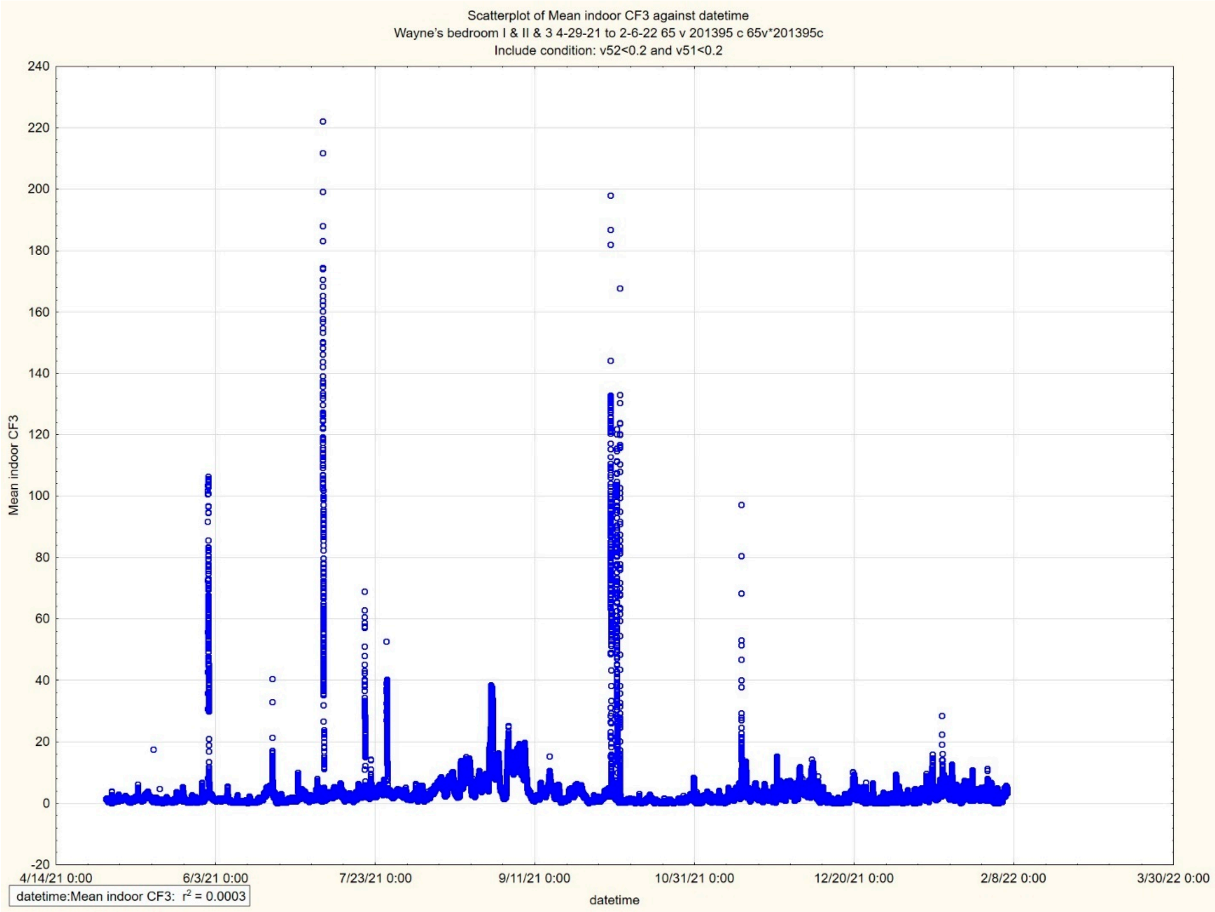

Figure S10. Mean indoor $PM_{2.5}$ ($\mu g/m^3$) using the ALT-CF3 algorithm from 4/29/21 to 2/6/22 at a site in Redwood City. All data with precision better than 20%.

The outdoor data is shown in Figure S11.

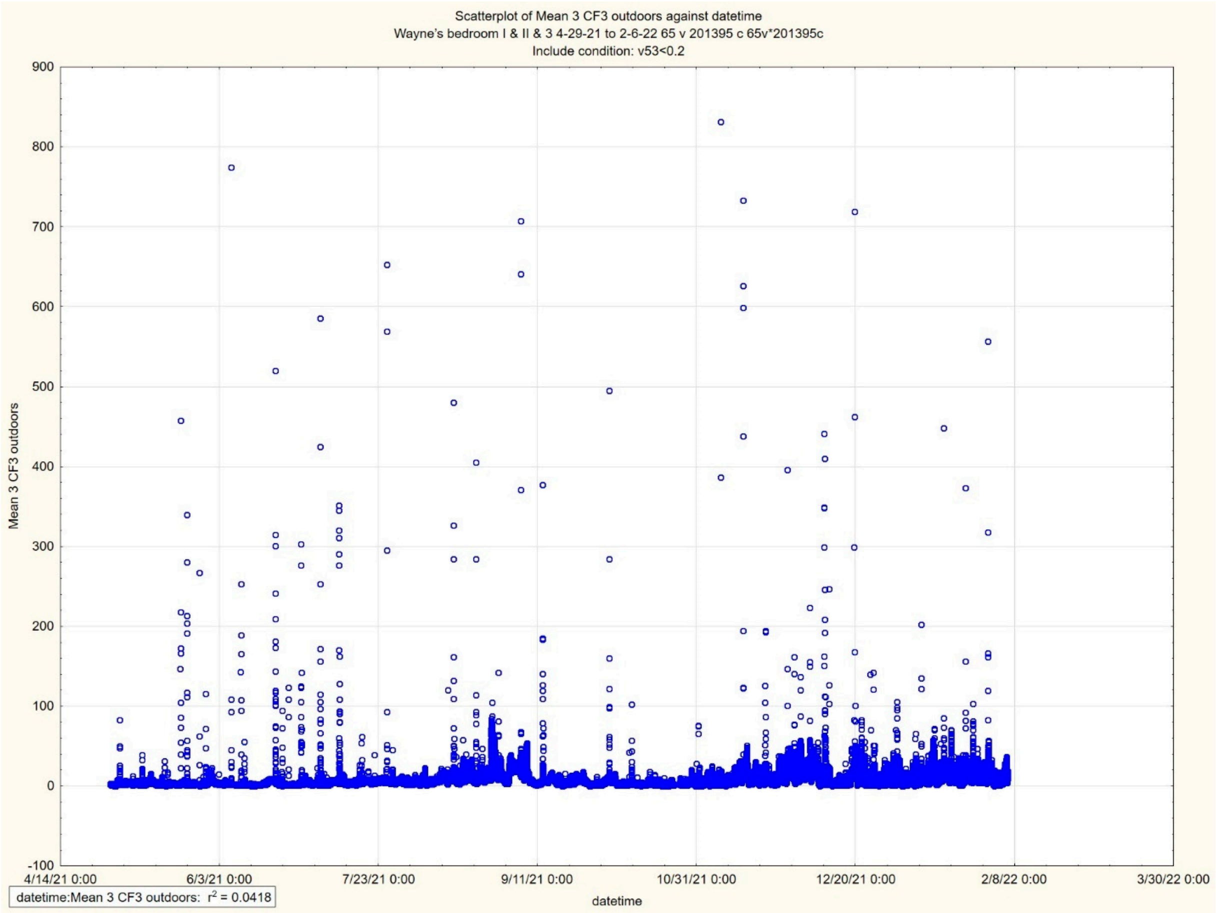

Figure S11. Outdoor data during the same period using the ALT-CF3 algorithm and again requiring precision to be better than 20%.

The best Spearman correlation (r_S) was for a lag time of 110 minutes (Table S9).

Table S9. Lag times for Redwood City site.

Lag times (minutes)	r_S
0	0.81550
60	0.84271
90	0.84757
100	0.84833
110	0.84853
120	0.84850
130	0.84829
150	0.84686

Limit of detection (LOD)

For monitor 1 indoors, the LOD for the Alt-CF3 algorithm was about 1.4 µg/m^3, corresponding to about 7500 (3%) of 200,000 observations being below the LOD. For the same monitor, the Plantower CF1 algorithm resulted in an estimated LOD of 4.75 µg/m^3, with 116,800 (58%) observations below the LOD.

In Figure S12, probably too many points fall into the Forbidden Zone below the estimated outdoor-penetrated concentration (red line) to accept the estimated infiltration factor of 0.4989. The estimated contribution of indoor-generated particles (0.189 µg/m^3) is very small, as could be expected for a home with only one nonsmoking occupant and little cooking.

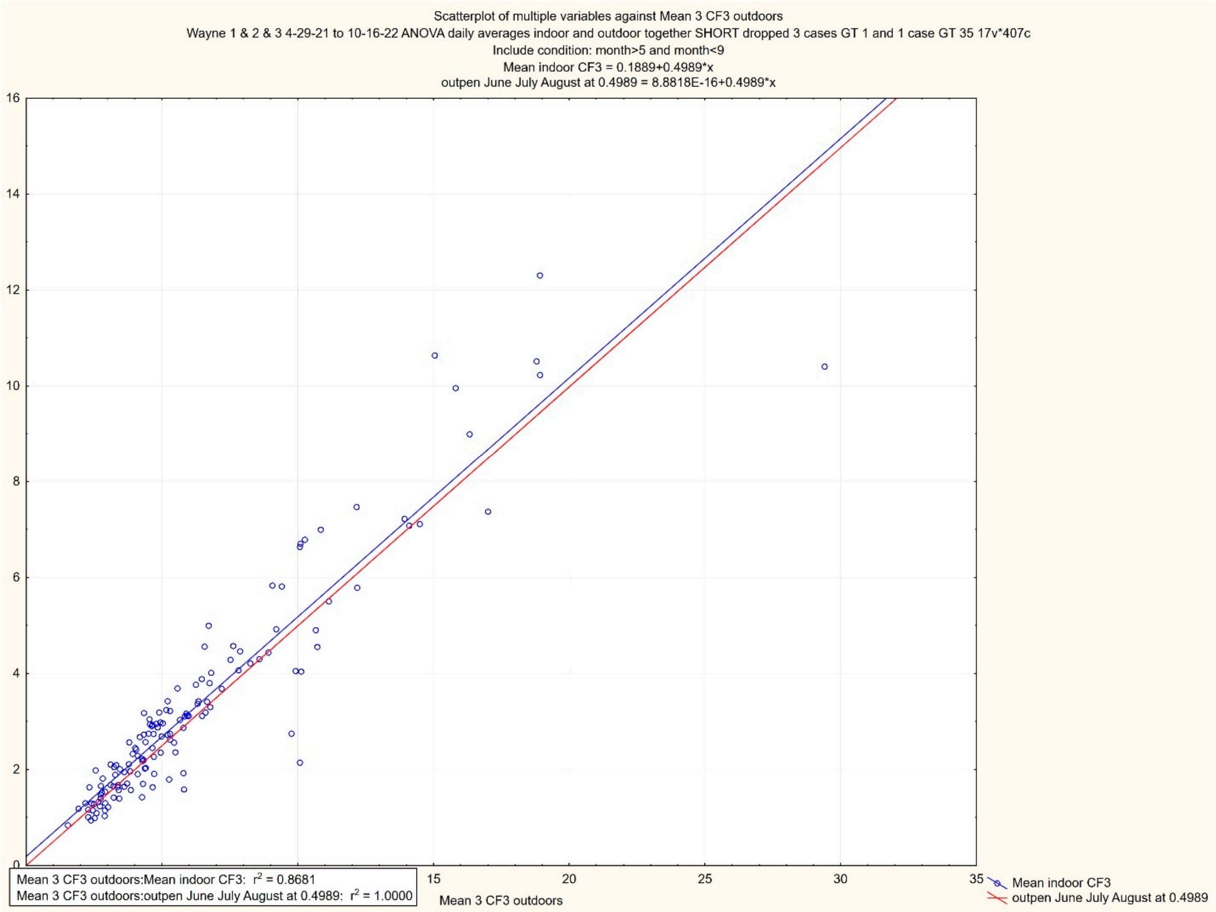

Figure S12. RCS approach to the June through August data with higher estimated infiltration factor of 0.4989.

All raw data were averaged by day; there were 281 days with matched pairs of daily averages in the data set. Indoor $PM_{2.5}$ was regressed on outdoor $PM_{2.5}$ (Figure S13), giving a slope of 0.367. These data support the conclusion that the infiltration factor was not constant, since there are >80 observations (>30%) in the forbidden zone.

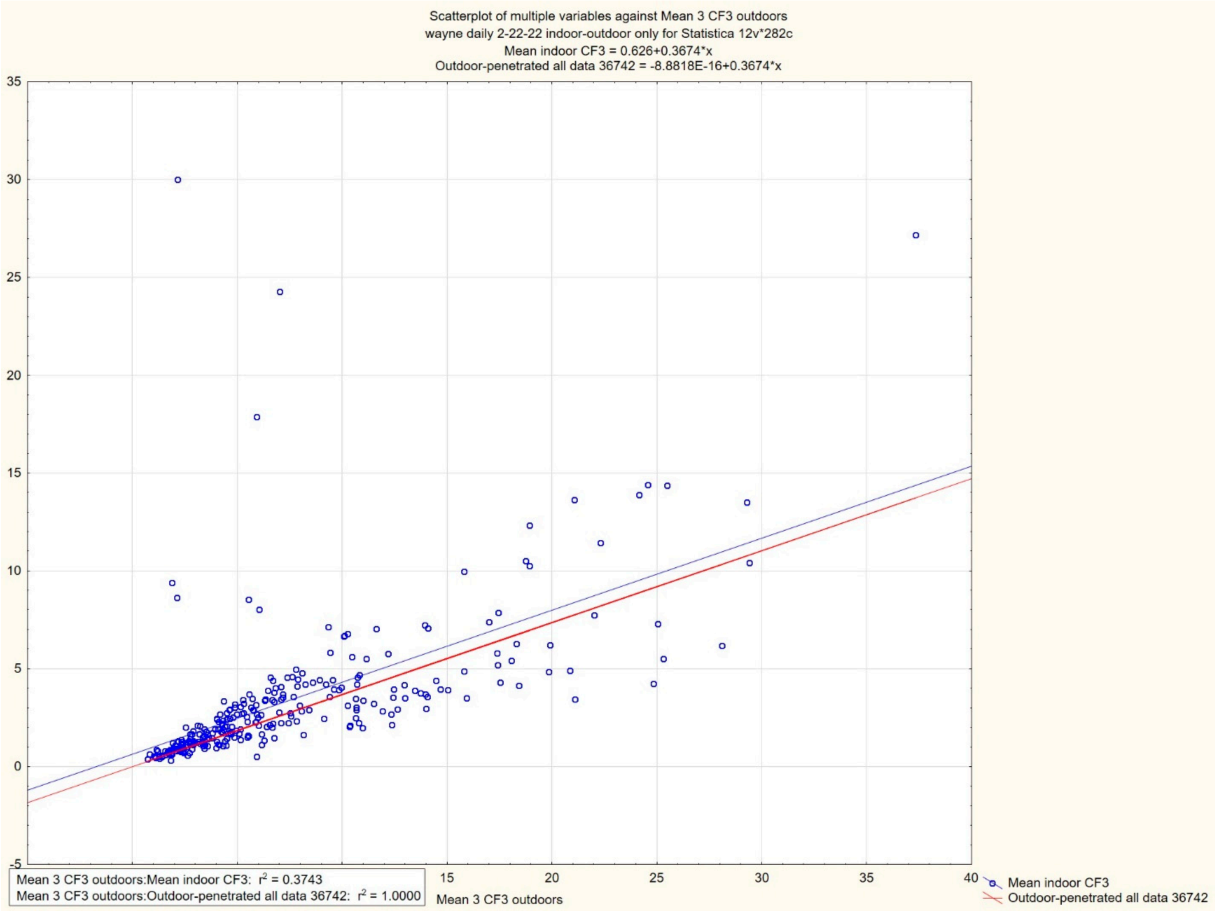

Figure S13. Regression of daily average indoor $PM_{2.5}$ on outdoor $PM_{2.5}$ using the CF3 algorithm--all data.

By inspection of individual months, the summer months appeared to have quite high infiltration factors. Therefore, they were deleted in favor of a 7-month regression (Figure S14). The regression on the 7-month dataset (221 days) resulted in only 7 of 221 (3%) observations found in the forbidden zone, cutting the percentage violating the Forbidden Zone from 30% to 3%. Moreover, all 7 observations hugged the Forbidden Zone boundary closely, suggesting that natural small daily variation in the infiltration factor

might account for the violations. The estimate of the infiltration factor was approximately 20% and the estimated $PM_{2.5}$ due to indoor activities was 1.145 $\mu g/m^3$.

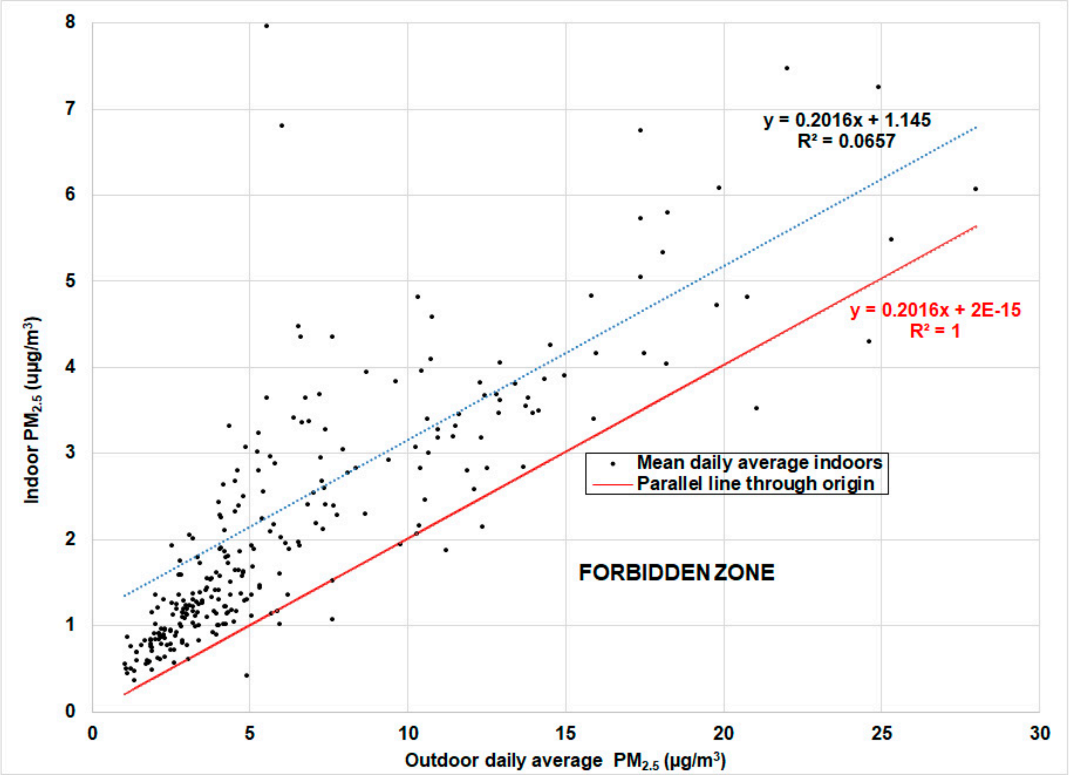

Figure S14. Regression of daily average indoor $PM_{2.5}$ on outdoor $PM_{2.5}$ using the CF3 algorithm—data for 7 months only.

Oakmont

Oakmont is a community within Santa Rosa, CA. Two indoor PA-II monitors 1 & 2 were set up on 7/23/19 and operated until 6/18/20; at that point, two additional PA-II monitors (3 & 4) were deployed. Monitors 1 & 4 were operated exclusively indoors, and monitor 3 (except for one month) outdoors for the next 19 months to February 14, 2022.

Quality assurance of the data was performed by setting a limit of 0.2 (20%) on the precision of both the indoor and outdoor measurements. This resulted in ~9% of the data being deleted. Also, several experiments resulting in elevated $PM_{2.5}$ for periods of several hours in one room of the house (which included at least one of the indoor monitors) were removed from consideration. This resulted in a further removal of ~0.6% of the data.

The regression of the indoor on outdoor $PM_{2.5}$ using the ALT-CF3 algorithm results in an intercept of 0.7026 $\mu g/m^3$ and a slope of 0.2789.

However, analysis of the monthly regressions showed a clear pattern of low infiltration factors (mean 0.11, SE 0.02) for the cool wet months of December through April (196 days), and high infiltration factors (mean 0.37, SE 0.04) for the warm dry months of May through November (316 days). The 7 months (May-November) with high infiltration factors and 5 months (December through April) with low infiltration factors matches up very well both with temperature and rainfall. The 6 months with highest average temperatures in Santa Rosa (59-66 °F) are May-October, while the 6 months with lowest temperatures (46-55 °F) occur in November-April. Rainfall is also limited to the November-April time frame, with the six months from May to October seldom experiencing rain. Doors and windows are more likely to be open when the weather is warm and dry, resulting in a higher infiltration factor in the warm months.

Therefore the regressions were rerun on those two periods (Table S10). The slope (infiltration factor) was 0.28 in the cool wet period, compared to 0.12 in the warm dry period. On the other hand, the intercept (mean indoor-generated $PM_{2.5}$) varied only slightly between about 0.8 and 1 μg/m^3. This is an important indication that a main assumption of the CRS theory, that indoor-generated $PM_{2.5}$ should not be correlated with outdoor $PM_{2.5}$, was upheld.

Table S10. Regressions of indoor $PM_{2.5}$ using the ALT-CF3 algorithm on outdoor $PM_{2.5}$ for all data and for the data in the two time periods.

	N obs	R^2 (adj)	Intercept	SE int.	z int.	p int.	Slope	SE slope	z slope	p slope
All data	333623	0.69	0.7026	0.0049	144	0	0.2789	0.00032	862	0
May-Nov	207606	0.73	0.9709	0.0070	140	0	0.2841	0.00038	754	0
Dec-April	126017	0.13	0.7829	0.0057	137	0	0.1233	0.00089	140	0

Fixing the infiltration factor for each of the two periods allows a direct calculation of the outdoor-penetrated data during each 2-minute observation. The difference between this value and the measured total indoor concentration provides an estimate of the indoor-generated $PM_{2.5}$ during that observation. The results of these calculations for the two time periods are provided in Table S11. During the cool wet months, the indoor-generated $PM_{2.5}$ outweighed the $PM_{2.5}$ entering from outside, amounting to almost 2/3 of the indoor concentration. During the warmer months, the indoor-generated contribution was about 1/3 of the total indoor $PM_{2.5}$.

Table S11. Observed indoor and outdoor $PM_{2.5}$ (µg/m^3) and estimated $PM_{2.5}$ due to indoor activities and infiltration of ambient air. Calculations based on raw data (2-minute average measurements).

	N obs	Mean	Median	Lower	Upper	Standard error
Cool wet months December-April						
Indoor monitor 1 CF3	126017	1.2	0.81	0.44	1.5	0.0050
Outdoor monitor 3 CF3	126017	3.8	2.2	1.1	4.1	0.0148
Outdoor-penetrated	126017	0.46	0.27	0.14	0.51	0.0018
Indoor-generated	126017	0.78	0.46	0.21	0.91	0.0047
Indoor precision	126017	0.071	0.062	0.029	0.105	0.00014
Outdoor precision	126017	0.057	0.047	0.022	0.083	0.00013
Warm dry months May-November						
Indoor monitor 1 CF3	207606	3.1	1.6	0.74	2.9	0.012
Outdoor monitor 3 CF3	207606	7.4	3.2	1.6	5.8	0.037
Outdoor-penetrated	207606	2.1	0.92	0.45	1.6	0.011
Indoor-generated	207606	0.97	0.52	0.10	1.3	0.0064
Indoor precision	207606	0.062	0.051	0.025	0.091	0.00010
Outdoor precision	207606	0.053	0.045	0.023	0.074	0.000087

These results were checked by calculating daily averages for the two periods. For the 7-month warm dry period the estimated infiltration factor was 0.3098, about 6% higher than the estimate of 0.2841 for the raw data (Figure S15). The forbidden region contains about 5% (~15 days out of 316) of the data.

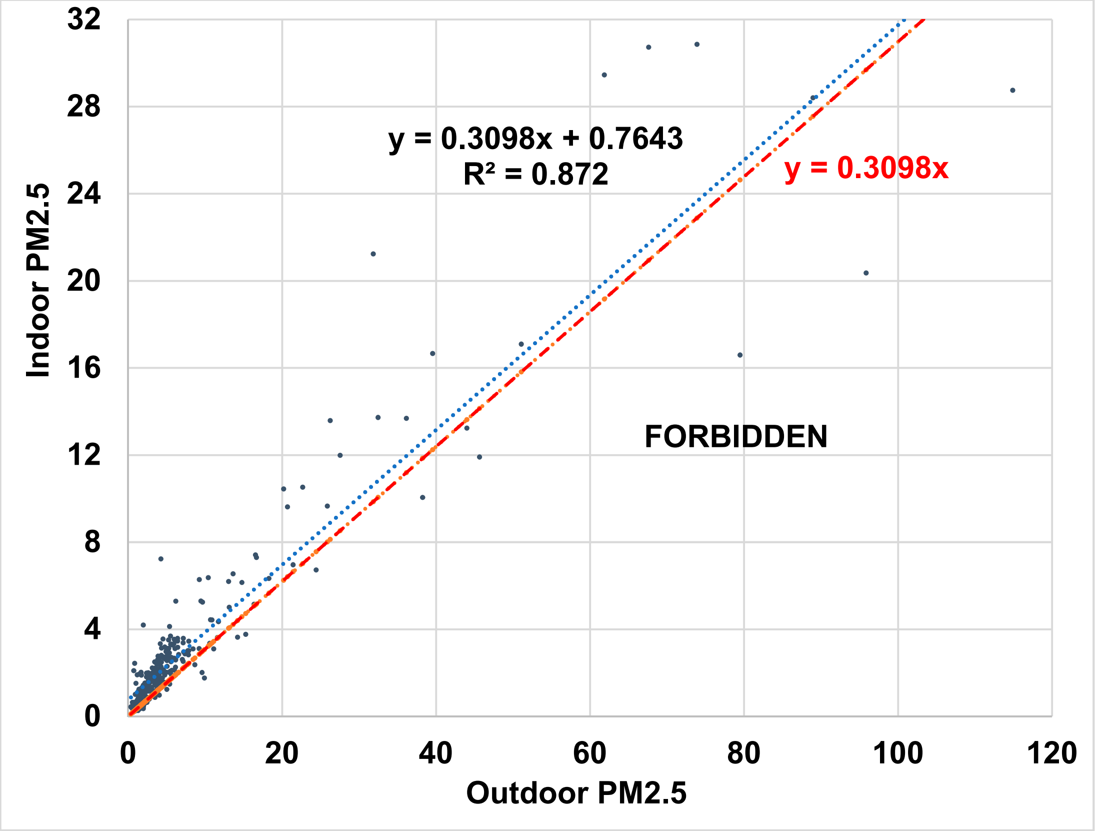

Figure S15. Regression of daily average indoor $PM_{2.5}$ on outdoor $PM_{2.5}$ during the 7 warm dry months.

For the cool wet period, the slope was about 0.20 for the daily averages compared to 1.2 for the 2-minute averages (Figure S16). The forbidden region contained about 15 (8%) of 196 daily averages.

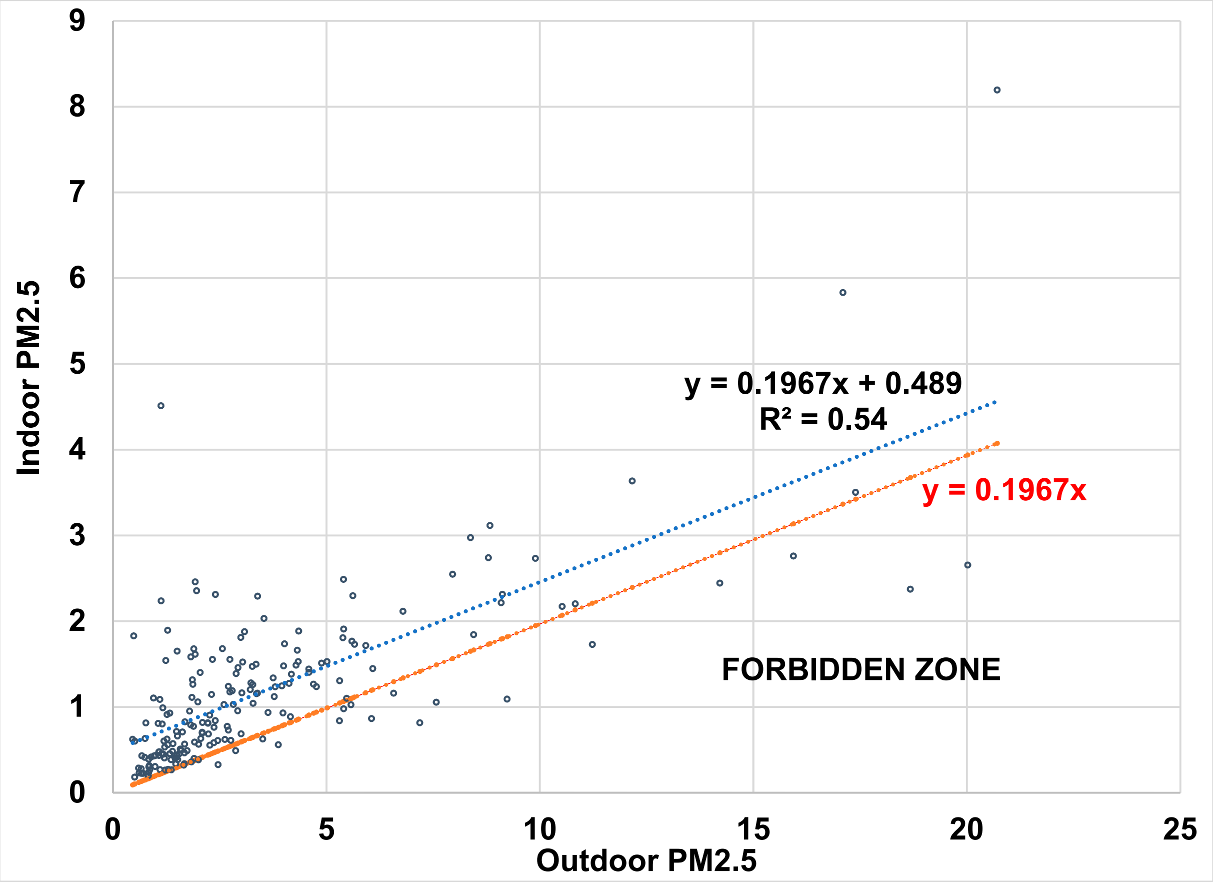

Figure S16. Regression of daily average indoor $PM_{2.5}$ on outdoor $PM_{2.5}$ during the 5 cool wet months.

The highest Spearman correlation of indoor and outdoor PM2.5 occurred near a lag time of 75 minutes (Table S12).

Table S12. Lag times

	Mean 3 $PM_{2.5}$ CF3 OUT
Indoor CF3	0.789
lag 45 minutes	0.804
lag 1 hour	0.8059
lag 75 minutes	0.8062
lag 90 minutes	0.8057
lag 2 hours	0.802
lag 150 minutes	0.797

Alexander Avenue

In Figure S17, too many points fall into the Forbidden Zone below the estimated outdoor-penetrated concentration (red line) and therefore we cannot accept the estimated infiltration factor of 0.2437.

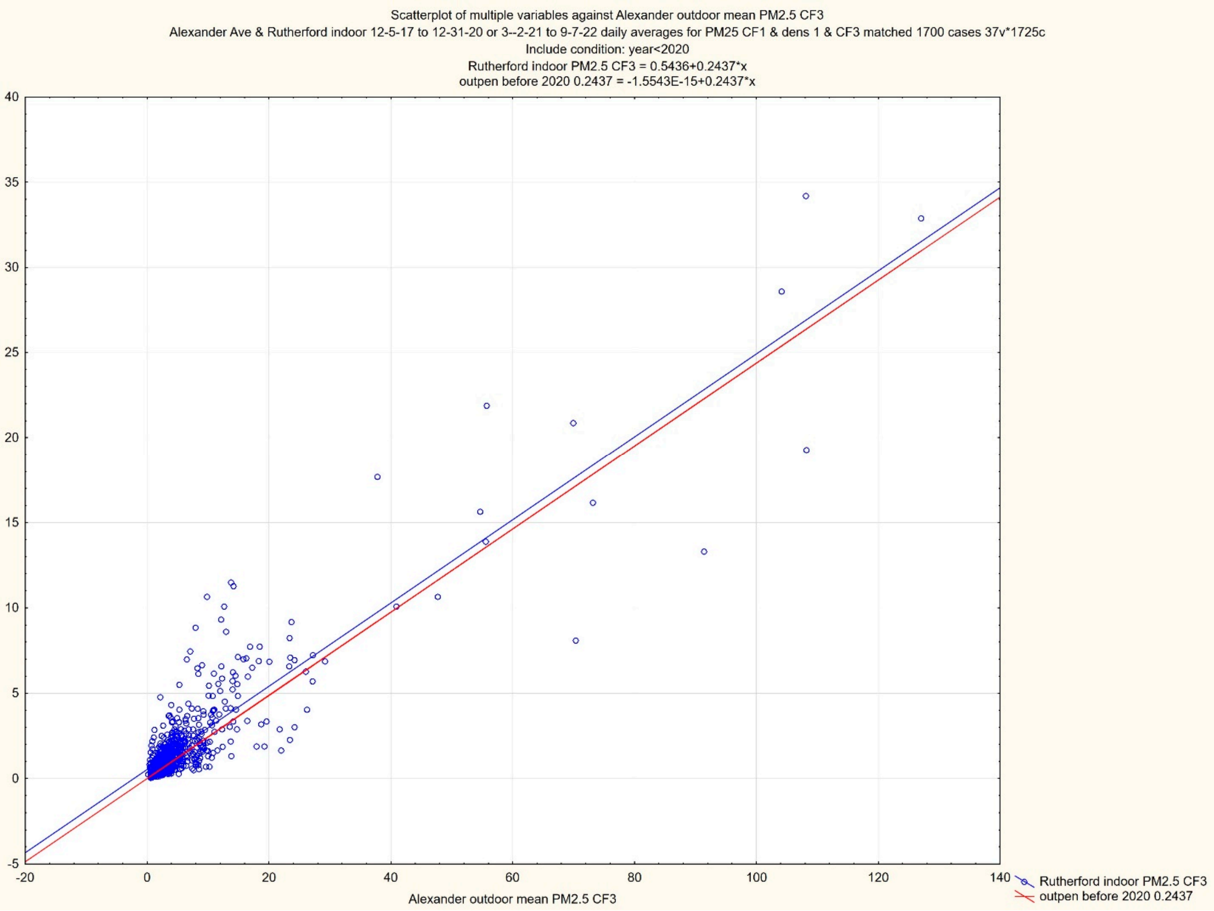

Figure S17. RCS approach to the period before 2020 with higher estimated infiltration factor of 0.2437.

Adjustment to the method of calculating the LOD.

For the Alexander Avenue site, the method of calculating the LOD using batches of 100 failed. Many batches of 100 at concentrations much higher than the expected LOD had >5 mean/SD ratios <3. Yet many batches had less than 5 ratios <3. We decided to go to batches of 1000, and seek the highest concentration with 50 ratios <3. This resulted in smoothing out the function very well and a clear LOD was found at 0.97 ug/m3.

Outer Sunset (San Francisco)

Table S13 shows the indoor $PM_{2.5}$ concentrations at the Outer Sunset 38th and SF site were relatively high, averaging 16.3 µg/m^3, while indoor sources averaged 14.2 µg/m^3, or 87% of the total.

Table S13. Summary Statistics for Outer Sunset 38th & Judah, SF, 1-14-22

Statistic	Outdoor Air (µg/m^3)	Indoor Air (µg/m^3)	Outdoor-Penetrated (µg/m^3)	Indoor-Generated µg/m^3
Mean	8.832	16.274	2.438	14.182
Median	5.346	10.268	1.476	7.896
Std. Dev.	16.073	17.626	4.436	17.113
Size	343	343	343	335

References

1. Wallace, L.A., Zhao, T., Klepeis, N.R. 2022 Indoor contribution to PM2.5 exposure using all PurpleAir sites in Washington, Oregon, and California.*Indoor Air* 32: (9) 13105. https://onlinelibrary.wiley.com/doi/abs/10.1111/ina.13105.

2. Wallace, L. Zhao, T. and Klepeis, N.E.. Calibration of PurpleAir PA-I and PA-II monitors using daily mean PM2.5 concentrations measured in California, Washington, and Oregon from 2017 to 2021. *Sensors* **2022, 22**, 4741. https://doi.org/10.3390/ s22134741 https://pubmed.ncbi.nlm.nih.gov/35808235/

3. Wallace, L. Intercomparison of PurpleAir Sensor Performance over Three Years Indoors and Outdoors at a Home: Bias, Precision, and Limit of Detection Using an Improved Algorithm for Calculating $PM_{2.5}$. *Sensors* **2022**, *22*, 2755. https://doi.org/10.3390/s22072755

4. Wallace, L., Bi, J., Ott, W.R., Sarnat, J.A. and Liu, Y. (2021) Calibration of low-cost PurpleAir outdoor monitors using an improved method of calculating $PM_{2.5}$. *Atmospheric Environment*, 256 (2021) 118432. https//doi.org/10.1016/j.atmosenviron.2021.118432 https://www.sciencedirect.com/science/article/abs/pii/S135223102100251X

5. Bi, J., Wallace, L., Sarnat, J.A. and Liu, Y. (2021). Characterizing outdoor infiltration and indoor contribution of $PM_{2.5}$ with citizen-based low-cost monitoring data. *Environmental Pollution 276:*116793. https://pubmed.ncbi.nlm.nih.gov/33631689/

6. Wallace, L.A.; Wheeler, A.; Kearney, J.; Van Ryswyk, K.; You, H.; Kulka, R.; Rasmussen, P.;

Brook, J.; Xu, X. Validation of continuous particle monitors for personal, indoor, and outdoor exposures. *J Expos Sci & Environ Epidemiol* **2010,** *21*, 49-64. https://www.nature.com/articles/jes201015

7. Robinson, D. Accurate, Low Cost $PM_{2.5}$ Measurements Demonstrate the Large Spatial Variation in Wood Smoke Pollution in Regional Australia and Improve Modeling and Estimates of Health Costs. *Atmosphere* **2020**, *11*(8), 856; **https://doi.org/10.3390/atmos11080856**

8. Barkjohn K, Gantt B, Clements AL. Development and application of a United States wide correction for $PM_{2.5}$ data collected with the PurpleAir sensor. Atmos Meas. Techniques **2020**;*4*(6):10.5194. https://doi.org/10.5194/amt-2020-413

9. Liang Y, Sengupta D, Campmier MJ, Lunderberg DM, Apte JS, Goldstein, A. Wildfire smoke impacts on indoor air quality assessed using crowdsourced data in California. _ Proc National Academy Sciences **2021**;118(36): e2106478118. https://www.pnas.org/doi/full/10.1073/pnas.2106478118.
